# Ultrasound-Guided Percutaneous Electrical Nerve Stimulation (PENS) as an Adjunct to a Multimodal Physical Therapy Program for Postoperative Shoulder Pain: A Randomized Clinical Trial

**DOI:** 10.3390/healthcare14111471

**Published:** 2026-05-26

**Authors:** Mario J. Abril-Serván, Fernando García-Sanz, Adrián Cases-Sebastia, Jorge Rodríguez-Jiménez, Gracia María Gallego-Sendarrubias, Joshua A. Cleland, José L. Arias-Buría

**Affiliations:** 1 Escuela Internacional de Doctorado, Universidad Rey Juan Carlos, 28008 Alcorcón, Spain; m.abrils@alumnos.urjc.es; 2 Department of Physical Therapy, Clínica CEMTRO, 28035 Madrid, Spain; fernando.garcia@clinicacemtro.com (F.G.-S.); adriancasese@gmail.com (A.C.-S.); 3 Department of Physical Therapy, Occupational Therapy, Physical Medicine and Rehabilitation, Universidad Rey Juan Carlos, 28922 Alcorcón, Spain; joseluis.arias@urjc.es; 4 Physiotherapy and Health Research Group (FYSA), Faculty of Health Sciences—HM Hospitals, University Camilo José Cela, 28692 Madrid, Spain; gmgallego@ucjc.edu; 5 Instituto de Investigación Sanitaria, HM Hospitals, 28015 Madrid, Spain; 6 Doctor of Physical Therapy Program, Department of Rehabilitation Sciences, Tufts University School of Medicine, Tufts University, Boston, MA 02111, USA; joshua.cleland@tufts.edu

**Keywords:** shoulder, suprascapular nerve, axillary nerve, percutaneous electrical nerve stimulation

## Abstract

**Highlights:**

**What are the main findings?**

- Adding two sessions of ultrasound-guided PENS to manual therapy/exercise improved postoperative shoulder pain and disability.
- The PENS group showed greater improvements in some strength and range of motion measures, but the practical clinical relevance of these differences remains to be confirmed.

**What are the implications of the main findings?**

- PENS targeting the suprascapular and axillary nerves may be a useful adjunct to multimodal rehabilitation after shoulder arthroscopy.
- Future trials should confirm the optimal PENS dosage, include sham controls, and assess longer-term outcomes.

**Abstract:**

Background/Objectives: Arthroscopic shoulder surgery is associated with postoperative pain and loss of function. Percutaneous electrical nerve stimulation (PENS) may serve as an effective adjunct to postoperative rehabilitation. This randomized clinical trial examined whether adding ultrasound-guided PENS to a multimodal rehabilitation program improves pain, disability, pressure pain sensitivity, shoulder range of motion, and muscle strength in individuals with postoperative pain following shoulder arthroscopy. Methods: A randomized, parallel-group clinical trial (registry: NCT06331871) was conducted. Seventy patients who had undergone shoulder arthroscopy were randomized to receive manual therapy/exercise alone (*n* = 35) or manual therapy/exercise/PENS (*n* = 35). All participants received the multimodal program including manual therapy and exercises four weeks after surgery for a duration of 12 weeks (five sessions/week). Those allocated to the PENS group also received two sessions (once per week) of ultrasound-guided PENS targeting the suprascapular and axillary nerves. Pain intensity (Numeric Pain Rating Scale (NPRS)) and disability (Disabilities of the Arm, Shoulder and Hand (DASH)) were the primary outcomes, whereas function (Shoulder Pain and Disability Index (SPADI)), pressure pain threshold (PPT), isometric strength, and shoulder range of motion (ROM) were secondary outcomes. Pain, PPT, strength, and ROM were assessed before and after treatment, and at 1 and 3 months. Disability and function were assessed at baseline and 3 months after treatment. Results: Patients receiving PENS showed greater improvements in shoulder pain (F_2.72, 182.32_ = 7.76, *p* = 0.007, η^2^*p* = 0.10), disability (F_1, 68_ = 5.63, *p* = 0.020, η^2^*p* = 0.08), function (F_1, 68_ = 4.15, *p* = 0.046, η^2^*p* = 0.02) and PPT over the infraspinatus muscle (F_3.20, 217.28_ = 2.93, *p* = 0.032, η^2^*p* = 0.04) than those receiving manual therapy/exercise alone. No between-group differences were observed for PPT at the deltoid or tibialis anterior muscles. The PENS group also showed greater improvements in some, but not all, measures of shoulder strength and range of motion; however, the effect sizes were small and the clinical relevance of these differences remains uncertain. Conclusions: Adding ultrasound-guided PENS targeting the suprascapular and axillary nerves to a multimodal physical therapy program resulted in greater improvements in pain, disability, and shoulder-specific function, with limited additional benefits for some measures of strength and range of motion, compared with physical therapy alone, in individuals with postoperative shoulder pain. However, many of the lower-bound estimates of the 95% confidence interval did not surpass the minimal clinically important difference. Therefore, the clinical relevance of the results should be considered with caution.

## 1. Introduction

Shoulder arthroscopy is one of the most commonly performed surgical procedures to manage rotator cuff tears and shoulder instability injuries. Despite refinements in surgical techniques [1], postoperative pain and functional limitations remain common and may delay functional recovery [2]. As a substantial proportion of patients report persistent pain or restricted range of motion [3] there is a need to identify effective postsurgical rehabilitation strategies [4,5,6].

Conventional postoperative pain management after shoulder arthroscopy typically includes opioid medication and in some cases an interscalene nerve block [7,8,9]. Although these approaches may be effective, they may involve adverse effects and are not always sufficient to optimize rehabilitation outcomes. This has increased interest in adjunctive approaches such as selective suprascapular or axillary nerve blocks and peripheral electrical stimulation, both of which may reduce opioid requirements and facilitate early postoperative recovery [10].

Physical therapy includes interventions targeting the peripheral and central nervous systems, such as electrical nerve stimulation, and are commonly applied for managing chronic pain. Transcutaneous electrical nerve stimulation (TENS), i.e., application of electrical current through electrodes, has been used for over 50 years to treat chronic pain, although its effectiveness is based on low-to-moderate-quality evidence [11].

Nevertheless, TENS may provide modest benefits for managing postoperative shoulder pain [12]. Percutaneous application of electrical current through solid needles (PENS) is being proposed as a novel therapeutic approach for managing musculoskeletal chronic pain [13]. Plaza-Manzano et al. found low-quality evidence supporting a positive benefit in pain and function for applying PENS on people with musculoskeletal pain conditions [14]. This review included studies using PENS targeting muscle, tendon or ligament tissues [14]. A scoping review found a small number of studies investigating PENS specifically targeting nerve tissues [15]. More recently, Mogedano-Cruz et al. observed limited evidence for the use of PENS targeting nerve tissue in peripheral neuropathies and encouraged future studies to investigate the use of this therapy as a treatment tool in physical therapy practice [16].

Postoperative pain after shoulder arthroscopy represents an ideal pathophysiological profile for the application of PENS since it is characterized by acute inflammation and central excitability changes [17]. Caballero-López et al. recently showed that combining ultrasound-guided PENS with physical therapy enhances analgesic effects and improves functional outcomes in painful postoperative conditions such as anterior cruciate ligament (ACL) reconstruction [18]. A case report where the application of PENS targeting the axillary nerve improved pain and pain-related disability in a patient with persistent postoperative pain after reverse total shoulder arthroplasty has recently been published [19].

To date, no randomized clinical trial has examined the clinical and neurophysiological effects of PENS in individuals suffering from postoperative pain after shoulder arthroscopy.

The purpose of this randomized clinical trial was to evaluate the effectiveness of adding ultrasound-guided PENS targeting the suprascapular and axillary nerves to a multimodal physical therapy program in individuals with postoperative pain following shoulder arthroscopy. We hypothesized that adding PENS would result in superior clinical, functional, and neurophysiological outcomes than application of physical therapy alone in people with postoperative pain after shoulder arthroscopy.

## 2. Materials and Methods

### 2.1. Study Design

We conducted a prospective, randomized, clinical trial registered with ClinicalTrials.gov (NCT06331871) and reported in accordance with the Consolidated Standards of Reporting Trials (CONSORT) extension for clinical trials [20]. The primary endpoint was three-month changes in pain intensity and pain-related disability. Secondary outcomes included shoulder-specific function, range of motion, isometric strength, and pressure pain sensitivity. No substantial changes were made to the methods after trial commencement.

### 2.2. Participants

Consecutive subjects who had undergone arthroscopic shoulder surgery at a single medical center (Clinica CEMTRO, Madrid, Spain) from March 2024 to March 2025 were screened for eligibility criteria. The specific inclusion criteria were: (1) aged over 18 years, (2) shoulder surgery within the previous four weeks, (3) experiencing shoulder pain after surgery, (4) experiencing limited joint mobility, and (5) had not received physical therapy treatment between surgery and their participation in the study. Participants were excluded if: (1) they had received previous neck/shoulder surgery, (2) had neurological disorders affecting upper-extremity motor control, (3) presence of metabolic disorders or any inflammatory disease, (4) were receiving medication treatment with antiplatelet agents, (5) had received corticosteroid or local anesthetic injections in previous years, or (6) presented with belonephobia or electrophobia (contraindications to the application of PENS).

### 2.3. Randomization and Blinding

Patients were randomly assigned to receive physical therapy alone or physical therapy plus PENS using a computer-generated block randomization table with a 1:1 allocation ratio using the Epidat software (version 4.2; Consellería de Sanidade, Xunta de Galicia, Santiago de Compostela, Spain) created by a statistician not involved in the trial. Individual and sequentially numbered index cards with the random assignment were folded and placed in sealed opaque envelopes. After completion of the baseline examination, the treating therapist opened the next sequential envelope and proceeded with treatment according to the allocation assignment. Because of the nature of the interventions, participants and treating therapists were not blinded to group assignment. The outcome assessor remained blinded to treatment allocation throughout follow-up.

### 2.4. Multimodal Physical Therapy Intervention

**Figure 1:**
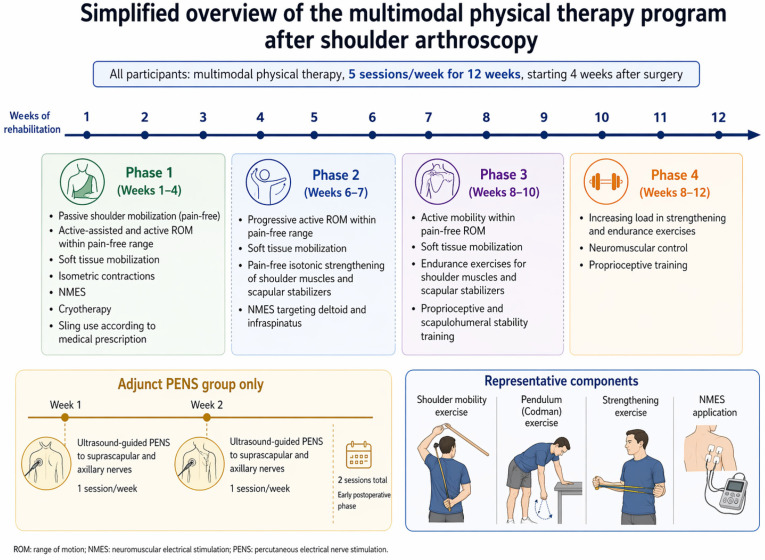
Simplified overview of the multimodal physical therapy program after shoulder arthroscopy. ROM: Range of motion; NMES: neuromuscular electrical stimulation; PENS: percutaneous electrical nerve stimulation.

All participants initiated a multimodal physical therapy treatment protocol four weeks after surgery (early mobilization). The program lasted 12 weeks (5 sessions/week) including manual therapy/exercises individually structured according to the biological phase of tissue healing and surgery characteristics. A multimodal evidence-based manual therapy program including joint mobilizations of the shoulder, soft-tissue mobilization techniques, neuromuscular electrical stimulation (NMES), strengthening, endurance, and proprioceptive exercises was also applied (Figure 1 and Supplementary Table S1) [21,22].

### 2.5. Ultrasound-Guided Percutaneous Nerve Stimulation

Participants assigned to physical therapy plus PENS received two ultrasound-guided sessions (one per week) applied to the suprascapular and axillary nerve as part of the rehabilitation program. Ultrasound guidance was used to enhance accurate localization of the neural structures and improve procedural safety [23]. Participants were positioned in side-lying position, with the affected upper limb relaxed alongside the body. Using a Versana Premier ultrasound system (GE HealthCare, Chicago, IL, USA) equipped with a 12 L linear transducer (1–18 MHz), the therapist identified both the suprascapular and the axillary nerve in the symptomatic shoulder.

**Figure 2:**
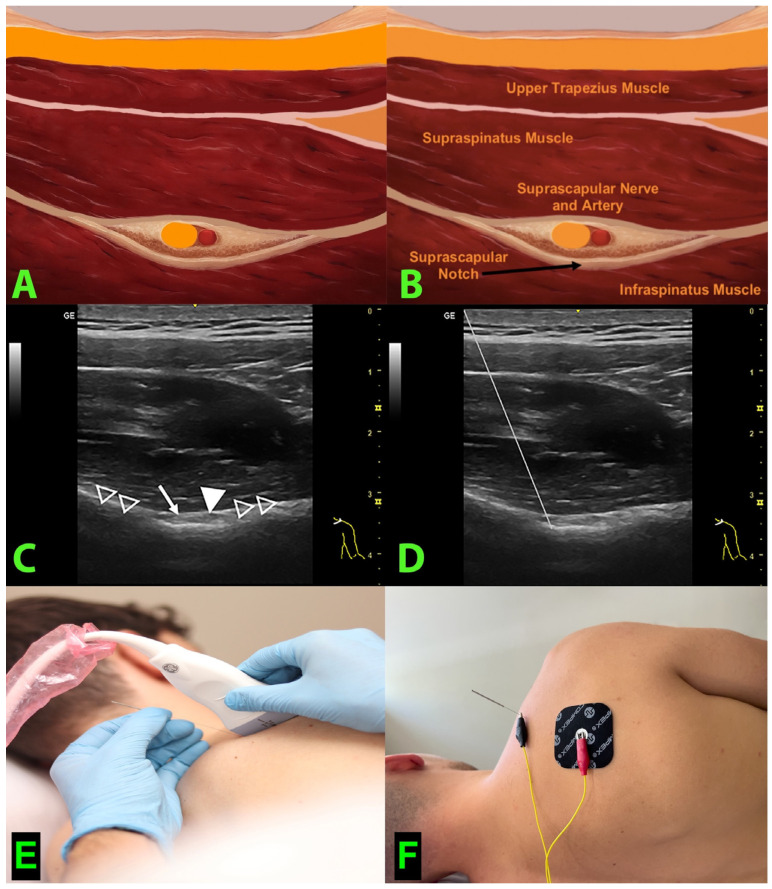
(**A**) Ultrasound-guided identification and percutaneous electrical nerve stimulation (PENS) of the suprascapular nerve (yellow) located lateral to the suprascapular artery (red) at the suprascapular notch. (**B**) The upper trapezius muscle is the most superficial, overlying the supraspinatus muscle. The suprascapular notch is located at the deepest part of the suprascapular fossa, where the nerve and artery course adjacent to one another before entering the supraspinous fossa. (**C**) Ultrasound view of the suprascapular fossa; the bony floor (open arrow) outlines the fossa and guides identification of the suprascapular nerve within the fascial plane. The suprascapular nerve (white arrow) and suprascapular artery/vein complex (arrowhead) can be visualized. (**D**) Ultrasound view of the needle path (white line) approaching the suprascapular nerve. (**E**) Ultrasound-guided needle insertion targeting the suprascapular nerve. (**F**) Application of PENS on the suprascapular nerve with the needle electrode and reference electrode coupled to channel one (yellow) of the ITO ES-160 device.

**Figure 3:**
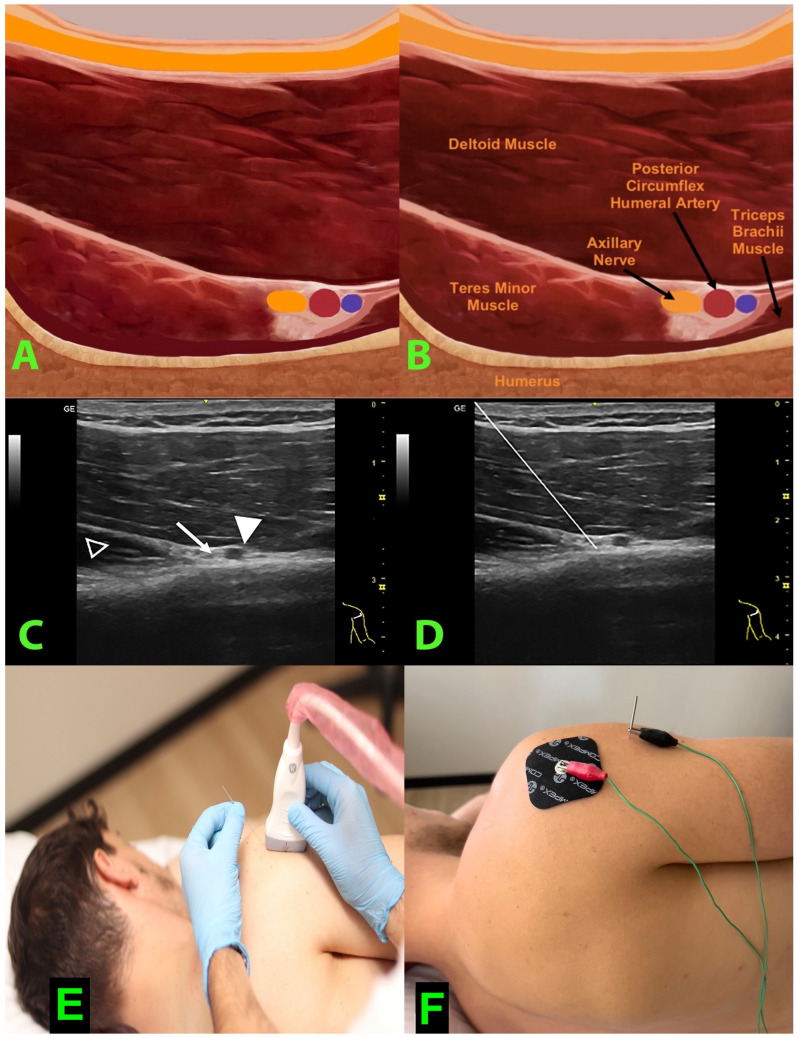
(**A**) Ultrasound-guided identification and percutaneous electrical nerve stimulation (PENS) of the axillary nerve (yellow) traveling through Velpeau’s quadrilateral space alongside the posterior circumflex humeral artery where it exits and runs around the surgical neck of the humerus toward the deep aspect of the deltoid muscle. (**B**) The axillary nerve is placed between the teres minor and the long head of the triceps brachii muscles. The posterior circumflex humeral artery (red) is visualized adjacent to the nerve. The deltoid muscle appears as the most superficial layer, followed by the deeper muscular boundaries of the quadrangular space. (**C**) Ultrasound view of the quadrangular space; the axillary neurovascular bundle is identified adjacent to the surgical neck of the humerus. The axillary nerve (white arrow) and posterior circumflex humeral artery (arrowhead) can be visualized. (**D**) Ultrasound view of the needle path (white line) approaching the axillary nerve. (**E**) Ultrasound-guided needle insertion targeting the axillary nerve on a real patient. (**F**) Application of PENS on the axillary nerve showing the needle electrode and reference electrode coupled to channel two (green) of the ITO ES-160 device.

The suprascapular nerve was identified at the suprascapular notch, within the supraspinous fossa, running beneath the supraspinatus muscle and the trapezius muscles (Figure 2A,B). The axillary nerve was identified at the superior border of the quadrilateral (Velpeau’s) space, between the teres minor and the long head of the triceps, where it travels with the posterior circumflex humeral artery (Figure 3A,B). After confirming the target landmarks under ultrasound (suprascapular nerve, Figure 2C; axillary nerve, Figure 3C), the skin was disinfected with 2% aqueous chlorhexidine (Lainco, Rubí, Barcelona, Spain). The objective was to position the needle tip adjacent to, but outside, the neural sheath of the suprascapular (Figure 2D) or axillary (Figure 3D) nerve, without entering the nerve. A solid filiform needle (0.30 × 50 mm or 0.30 × 60 mm; AguPunt, Barcelona, Spain) was inserted using an in-plane approach under continuous ultrasound guidance toward the target nerve (suprascapular, Figure 2E; axillary, Figure 3E), while carefully avoiding the suprascapular vessels or the posterior circumflex humeral artery. If neurological symptoms were provoked or needle-tip visualization was lost, the needle was immediately withdrawn.

To confirm that the selected nerve had been appropriately targeted, a Pointer Plus stimulator (Goldberg International Enterprises Ltd., Kowloon, Hong Kong) was applied over the needle to deliver 2 or 3 electrical pulses at a frequency of 10 Hz and 2.5–3 mA. Needle placement was considered accurate when this brief stimulation evoked a visible contraction in muscles innervated by the corresponding nerve. Once correct placement had been verified, the needles were connected to an electrostimulator (ITO ES-160 device, Ito Co., Ltd., Tokyo, Japan), and biphasic electrical stimulation was delivered at 2 Hz with a pulse width of 250 μs for 30 min. The intensity of the current was increased only until a visible, non-painful contraction of the innervated musculature occurred.

### 2.6. Primary Outcomes

The intensity of shoulder pain during the previous week was assessed with an 11-point numeral pain rating scale (NPRS) (0: no pain, 10: worst imaginable pain) [24] at baseline, after the last treatment session, and at a one- and three-month follow-up. The minimal clinically important difference (MCID) for the NPRS in people with shoulder pain has been estimated to be 1.1 points [25]. However, Michener et al. reported that a change of 2.2 points on the NPRS is to be considered the MCID in outpatient rehabilitation [26].

Pain-related disability was assessed at baseline and at three months with the validated Spanish version of the Disabilities of the Arm, Shoulder and Hand (DASH, score: 0–100 points) questionnaire [27]. The DASH includes 21 items which are answered on a 5-point Likert scale (1: no problem, no symptom, no impact, 5: impossible to do, extremely severe symptom, high impact) specific to the difficulties experienced during the previous week when performing physical activities. Five items evaluated the severity of pain symptoms, and four items assessed the effects of pain on social activities, work, and sleep [28]. The MCID for the DASH has been determined to be 11 points in patients with unspecific shoulder pain [29] and patients with calcific tendinitis of the rotator cuff [30]. Gummesson et al. found that changes in DASH fluctuate over time after upper-extremity surgery, but these authors reported that a 10-point difference could be considered the minimal important change [31]. Similarly, a recent review on different patient-reported outcome measures (PROMs) for shoulder pain determined that 1.5 points and 10 points can be considered as an estimated pooled MCID for NPRS and DASH, respectively [32].

### 2.7. Secondary Outcomes

Shoulder-specific pain and function were assessed at baseline and a 3-month follow-up with the Spanish version of the Shoulder Pain and Disability Index (SPADI) which includes 13 items, five on pain and eight on pain-related disability [33]. The MCID of the SPADI has been established to range between 8 and 13 points depending on population and context [34]. More recently, it has been reported that the MCID cutoff of the SPADI ranges from 14 to 20 points after rotator cuff repair [35] or shoulder arthroplasty [36].

Neurophysiological and functional outcomes were assessed at baseline, after the last session, and at one- and three-month follow-up periods by an assessor blinded to the treatment allocation of the subjects. Pressure pain thresholds (PPTs) were assessed with a mechanical algometer (FDK/FDN, Wagner Instruments, Greenwich, CT, USA). Participants were seated comfortably with the affected upper extremity supported and relaxed. Pressure was applied perpendicularly at a rate of approximately 1 kg/cm^2^/s over the following points: infraspinatus muscle (suprascapular nerve-related area), deltoid muscle (axillary nerve-related area), and tibialis anterior muscle (distant pain-free area). Each site was measured three times with a 30 s interval between tests, and the mean value was used for the main analysis. This procedure has been shown to have high reliability and sensitivity to change in patients with shoulder pain [37,38].

Isometric shoulder strength was measured with a portable hand-held dynamometer (ActivForce, Activbody, San Diego, CA, USA). The flexion/extension, abduction/adduction, and internal/external rotation force of affected shoulder was assessed using standardized positions. For each movement, the dynamometer was aligned with the force vector perpendicularly applied to the distal part of the arm while participants executed a 4 s maximal isometric contraction, three times, with the mean value used for analysis. The ActivForce device has been shown to have excellent intra- and inter-rater reliability for upper-limb strength assessment (intraclass correlation coefficients ranging from 0.93 to 0.98) and a Minimal Detectable Change (MDC) of approximately 5–10 N [39,40].

Finally, shoulder range of motion in flexion/extension, internal/external rotation, and abduction was evaluated using a universal goniometer following the standardized protocol of the American Academy of Orthopaedic Surgeons (AAOS). This method has been shown to have good-to-excellent intra-rater reliability (ICC ranging from 0.84 to 0.99) with an MDC of approximately 5° to 8°, supporting precise detection of clinically meaningful changes in surgical rehabilitation contexts [41,42,43].

No changes to the prespecified outcomes were made after trial commencement.

### 2.8. Sample Size Calculation

The sample size calculation was performed using G*Power 3.1 (Heinrich Heine University Düsseldorf, Düsseldorf, Germany) for a repeated-measures analysis of variance (ANOVA) (within–between interaction), assuming a medium effect size (f = 0.25), α = 0.05, power = 0.95, 2 groups, and 6 measurement points. This analysis yielded a minimum required total sample size of 28 participants (actual power = 0.954). A dropout percentage of 20% during follow-up was expected, so the sample was increased to at least 30 patients per group. No interim analyses or stopping rules were planned.

### 2.9. Statistical Analysis

Statistical analysis was performed using IBM SPSS v.29 (IBM Corp., Armonk, NY, USA) and Jamovi v2.6 (The jamovi project, Sydney, Australia) software and was conducted according to the intention-to-treat principle. Between-group comparisons at baseline were performed using independent-samples *t*-tests or Chi-square tests as needed. Normality was assessed using the Shapiro–Wilk test and Q–Q plots.

A repeated-measures ANOVA (ANOVA MR) was used to examine the effects of the intervention on each outcome variable separately. The model included two factors: a between-subjects factor (group: PENS vs. control) and a within-subjects factor (time: 6 measurement points—baseline, post-session 1, pre-session 2, post-session 2, 1-month follow-up, and 3-month follow-up). For disability (DASH) and shoulder-specific function (SPADI), which were only assessed at baseline and 3-month follow-up, a 2 × 2 ANOVA MR was applied. Both main effects (time and group) and their interaction (group × time) were examined. The sphericity assumption was assessed with Mauchly’s test; when violated, degrees of freedom were corrected using the Greenhouse–Geisser method. Homogeneity of variances was assessed with Levene’s test. For outcomes where this assumption was violated or where residual plots indicated heteroscedasticity, a Mixed Linear Model (MLM) was used as an alternative, reporting marginal R^2^ (R^2^m, variance explained by fixed effects) and conditional R^2^ (R^2^c, total variance explained). Post hoc pairwise comparisons were conducted with Bonferroni correction. The effect size was reported as partial eta squared (η^2^*p*), with values of 0.01, 0.06, and 0.14 representing small, medium, and large effects, respectively [44]. Statistical analyses were performed using IBM SPSS v.29 and jamovi v.2.6. The a priori sample size calculation was performed using G*Power 3.1 for a repeated-measures ANOVA (within–between interaction), assuming α = 0.05, power = 0.95, a medium effect size (f = 0.25), 2 groups, and 6 measurement points, yielding a minimum required sample size of 28 participants (actual power = 0.954). The repeated-measures ANOVA therefore included all six assessment points. For clarity of presentation, outcome data are reported at 4 representative time points in the tables (baseline, post-intervention, 1-month, and 3-month follow-up).

## 3. Results

### 3.1. Participants

**Figure 4:**
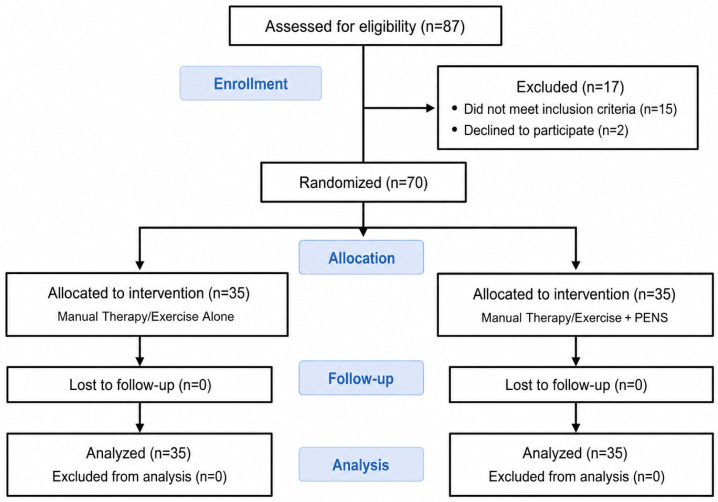
Participant flow through the randomized clinical trial.

Eighty-seven consecutive patients scheduled for shoulder arthroscopy surgery were screened for eligibility criteria. Seventy subjects (mean age (±SD): 50 ± 15.5 years, 35.7% female) satisfied the eligibility criteria, agreed to participate, and were randomized into manual therapy/exercise alone (*n* = 35) or manual therapy/exercise/PENS (*n* = 35) groups. No subjects were lost to dropout during the study. Figure 4 provides the flow diagram of patient recruitment and retention. Baseline features between groups were similar for all variables (Table 1). The trial ended as planned after the target sample size was achieved and all prespecified follow-up assessments were completed. No adverse events or unintended effects related to the interventions were reported in either group.

### 3.2. Clinical Outcomes

Sphericity assumptions were tested with Mauchly’s test; Greenhouse–Geisser corrections were applied where they were violated. Homogeneity of variances was assessed with Levene’s test; Mixed Linear Models (MLM) were used when they were violated.

The ANOVA MR revealed a significant main effect of time (ε = 0.54, F_2.72, 182.32_ = 125.77, *p* < 0.001, η^2^*p* = 0.65), a significant main effect of group (F_1, 68_ = 7.63, *p* = 0.007, η^2^*p* = 0.10), and a significant group × time interaction (F_2.72, 182.32_ = 7.76, *p* = 0.007, η^2^*p* = 0.10) for pain intensity (NPRS). Post hoc analyses revealed that the PENS group showed significantly greater reductions in pain compared with the control group post-session 1 (*p* < 0.001), pre-session 2 (*p* = 0.010), post-session 2 (*p* < 0.001), and at 1-month follow-up (*p* = 0.019), with no significant between-group difference at 3-month follow-up (*p* = 0.126) (Table 2). For disability (DASH), the ANOVA MR revealed a significant main effect of time (F_1, 68_ = 339.08, *p* < 0.001, η^2^*p* = 0.83) and a significant group × time interaction (F_1, 68_ = 5.63, *p* = 0.020, η^2^*p* = 0.08), with the PENS group showing greater reductions in disability at 3-month follow-up (Δ −13.3, 95%CI −23.3 to −3.3) compared with the control group (Table 2).

A significant group × time interaction was also observed for shoulder-specific function (SPADI: F_1, 68_ = 4.15, *p* = 0.046, η^2^*p* = 0.02), with the PENS group showing greater improvement at 3-month follow-up (Δ −13.9, 95%CI −25.9 to −1.9) compared with the control group (Table 2).

### 3.3. Neurophysiological Outcomes

The ANOVA MR revealed a significant main effect of time (ε = 0.64, F_3.20, 217.28_ = 72.53, *p* < 0.001, η^2^*p* = 0.52) and a significant group × time interaction (F_3.20, 217.28_ = 2.93, *p* = 0.032, η^2^*p* = 0.04) for PPT at the infraspinatus muscle. Post hoc analyses revealed greater improvements in the PENS group post-session 1 (*p* < 0.001), reflecting an acute hypoalgesic effect, which was not maintained at subsequent time points. No significant group × time interactions were observed for PPT at the deltoid muscle (F_3.08, 209.57_ = 2.13, *p* = 0.096, η^2^*p* = 0.03) or tibialis anterior muscle (F_5, 340_ = 3.88, *p* = 0.002 for time effect; group × time interaction not significant) (Table 3).

### 3.4. Physical Outcomes

The MLM analyses revealed significant group × time interactions for mean isometric strength in adduction (F_5, 340_ = 3.31, *p* < 0.001), internal rotation (F_5, 339_ = 2.82, *p* = 0.016), mean extension (F_5, 340_ = 4.69, *p* < 0.001), and maximum extension (F_5, 340_ = 7.10, *p* < 0.001), with the PENS group showing greater improvements from post-session 2 onwards. No significant group × time interactions were observed for mean flexion (F_5, 340_ = 1.26, *p* = 0.282), mean abduction (F_5, 340_ = 1.19, *p* = 0.314), or mean external rotation (F_5, 340_ = 0.48, *p* = 0.788) (Table 4).

The MLM analyses revealed significant group × time interactions for ROM in flexion (F_5, 340_ = 2.79, *p* = 0.017), extension (F_5, 340_ = 7.27, *p* < 0.001), and internal rotation (F_5, 340_ = 2.58, *p* = 0.026), with the PENS group showing greater improvements from post-session 2 onwards. No significant group × time interactions were observed for external rotation (F_2.18, 148.22_ = 1.59, *p* = 0.206), abduction (F_5, 340_ = 0.51, *p* = 0.771), or adduction (Table 5).

## 4. Discussion

This randomized clinical trial suggests that adding ultrasound-guided PENS directed at the suprascapular and axillary nerves to a multimodal physical therapy program may result in greater improvements in pain, disability, and shoulder-specific function after shoulder arthroscopy, with limited additional benefits for strength or range of motion. No intervention-related adverse events were reported.

### 4.1. Effects of PENS on Pain and Pain-Related Disability

The between-group differences exceeded the established minimal clinically important difference thresholds at some follow-up points; however, the confidence intervals encompassed the MCID values, indicating that the clinical relevance of these differences should be interpreted cautiously. Similarly, the between-group difference for pain-related disability was moderate and exceeded the MCID for DASH (10 points), but again the 95% CI confidence intervals included this score. Based on these findings, there may be a statistically significant benefit of adding ultrasound-guided PENS to manual therapy and exercises for optimizing postoperative recovery after shoulder arthroscopy; however, it cannot be determined if differences between groups are clinically relevant. Previous studies investigating the addition of needling interventions to exercise in individuals with shoulder pain conditions are conflicting. For instance, adding electro-acupuncture to an exercise program did not result in a positive effect in individuals with subacromial pain syndrome [45], yet adding electrical dry needling to multimodal physical therapy was effective in people with non-specific shoulder pain [46]. Similar heterogeneous results are reported for the inclusion of PENS into exercise programs in individuals with general shoulder pain. Valenzuela-Rios et al. found that adding four sessions of PENS targeting the axillary and suprascapular nerves to an exercise program did not result in superior clinical or psychological outcomes when compared to placebo PENS in people with subacromial pain syndrome [47]. Góngora-Rodríguez et al. recently reported that the combination of PENS and percutaneous electrolysis with exercise resulted in better clinical and functional outcomes compared to electrotherapy (TENS and ultrasound) and exercise in individuals with supraspinatus tendinopathy [48]. These discrepancies may be related to differences in patient populations, postoperative status, co-interventions, treatment dosage, and stimulation targets. For instance, Lewis et al. included people with full-thickness/massive irreparable rotator cuff tear [45], Góngora-Rodríguez et al. included patients with supraspinatus tendinopathy [48] and the current study included people with shoulder pain after an arthroscopic procedure. Therefore, comparisons across studies should be made with caution. Rather than indicating that one needling approach is superior to another, the current evidence suggests that treatment response is likely influenced by the interaction between clinical presentation, stage of recovery, and intervention parameters.

### 4.2. Effects of PENS on Muscle Strength and Range of Motion

Since PENS targets nerve tissue, it has been hypothesized that this intervention may facilitate better contraction of the muscles innervated by the targeted nerves and, thus, improvements in functional outcomes may be observed. This hypothesis is based on evidence suggesting the application of low frequencies (2 Hz) with an intensity producing muscle contraction has been shown to increase the electrical motor threshold and modify motor recruitment, although these physiological effects do not necessarily translate into clinically relevant hypoalgesia in healthy subjects [49].

Preliminary evidence suggests the application of PENS improves range of motion and muscle strength in the lower extremity (e.g., hip range of motion when targeting the sciatic nerve or knee range of motion when targeting the femoral nerve) [50,51,52]. In patients with postoperative shoulder pain, PENS may mitigate the early neuromuscular inhibition and impaired voluntary activation of the shoulder muscles commonly seen after the surgical procedure [53]. Our clinical trial observed that adding PENS resulted in limited but statistically significant benefits for some measures of isometric shoulder strength and range of motion. Specifically, significant group × time interactions were found for mean and maximum adduction, internal rotation, and extension strength, as well as for ROM in flexion, extension, and internal rotation, with the PENS group showing greater improvements from post-session 2 onwards. However, no significant between-group differences were observed for flexion, abduction, or external rotation strength, nor for external rotation or abduction ROM. These findings suggest that the effects of PENS on functional outcomes may be selective and direction-dependent, possibly related to the innervation pattern of the targeted nerves. The suprascapular nerve innervates the supraspinatus and infraspinatus muscles, while the axillary nerve innervates the deltoid and teres minor. The observed improvements in adduction, internal rotation, and extension—movements not primarily driven by these muscles—may reflect indirect neuromuscular facilitation or reduced pain inhibition rather than direct motor activation. Our results partially align with those of Góngora-Rodríguez et al. [48], who reported functional improvements following PENS in shoulder pain conditions, and contrast with Valenzuela-Ríos et al. [47], who found no superior functional outcomes compared to placebo PENS. These discrepancies may be explained by differences in dosage, targeted nerves, and the postoperative context of the current study. The clinical relevance of these functional improvements should nonetheless be interpreted cautiously, as effect sizes were small and between-group differences did not consistently exceed established MCID thresholds.

### 4.3. Effects of PENS on Pain Sensitivity

It has been argued that PENS promotes analgesia by reducing primary (peripheral mechanism) and secondary (central mechanism) hyperalgesia. In fact, current evidence has shown PENS has a mild-to-moderate effect on local (peripheral) and remote (central) hyperalgesia to pressure pain in individuals with chronic musculoskeletal pain, although this assumption is based on a small number of studies [54]. Since post-surgical pain has a pathophysiological profile characterized by acute inflammation, peripheral sensitization and central excitability, we investigated the hypoalgesic effects by assessing local and widespread sensitivity to pressure pain.

We observed an immediate and significant increase in PPT (e.g., hypoalgesic effect) at the infraspinatus muscle; however, there we no differences in PPTs at the deltoid and tibialis anterior muscles in people receiving PENS compared with those not receiving PENS. The results demonstrated an immediate local, but not distal, hypoalgesic effect, suggesting peripheral, but not central, desensitization. In fact, this effect was only significant after treatment, and not at the one- or 3-month follow-up periods. The presence of local hypoalgesia to pressure pain in the infraspinatus muscle may be explained by a decrease in neural firing at the spinal cord since this muscle is innervated by the suprascapular nerve. However, we did not observe this effect on the deltoid muscle, which is innervated by the axillary nerve. It has been shown that the effects of electrical current depend on the frequency of application, where the activation of the endogenous opioid receptors depends on the stimulation received. The stimulation parameters used in the current study (2 Hz, 250 μs) are within the range known to promote endogenous release of *β*-endorphins, serotonin and noradrenaline [55]. Therefore, it is possible that the neuroanatomy differences between the nerves (e.g., the axillary nerve is shorter and runs between more muscle layers anatomically than the suprascapular nerve) could lead to different responses with the identical nerve stimuli.

It should be noted that both groups experienced similar increases in PPT both locally (i.e., infraspinatus/deltoid muscles) and distally (i.e., tibialis anterior muscle), suggesting the multimodal protocol including manual therapy/exercise may have central hypoalgesic effects. It has been suggested that exercise-induced hypoalgesia might be peripherally (by modulating transduction, transmission and processing of noxious stimuli) and centrally (by a systemic activation of descending inhibitory pain pathways) mediated [56]. Similarly, it has been identified that manual therapy can potentially exert hypoalgesic effects that are both peripherally (moderate-quality evidence) and centrally mediated [57].

### 4.4. Limitations

The results of the current study should be considered according to the strengths and limitations of this trial. The major strengths include concealed allocation, use of blinded assessors, and intention-to-treat analyses. The potential limitations include the fact that participants were recruited from a single clinic, which may reduce the generalizability of the findings. Also, we did not include a wait-and-see or no-intervention group; therefore, we cannot determine if the changes observed in both groups can be attributed to interventions or simply the passage of time. The lack of participant and therapist blinding may have introduced expectation bias, particularly for self-reported outcomes such as pain intensity and disability. The outcomes were assessed only as mid-term effects (3-month follow-up); therefore, it cannot be determined whether the changes would be maintained at longer follow-up periods. Furthermore, the placebo effect of PENS cannot be determined as we did not have a group receiving sham PENS intervention [58]. The most appropriate dosage of PENS remains to be elucidated. In fact, subjects allocated to the PENS group received just two sessions at the beginning of the treatment protocol based on clinical experience, as no scientific data is available suggesting the most optimal dose or the frequency of this intervention; accordingly, it is unknown if a higher number and/or a greater frequency of sessions would result in larger differences. Finally, the inclusion of a variety of surgical procedures may have introduced inter-subject variability; however, this situation reflects daily clinical practice. Analgesic medication use was not systematically recorded and could not be controlled for as a covariate, which represents a relevant limitation given that pain intensity was a primary outcome. Since the sample size was calculated for the main between-group comparison, the study was not powered to perform subgroup analyses by type of surgery. Future studies should consider stratified randomization or planned subgroup analyses according to surgical procedure. Additionally, no formal prespecified sensitivity analysis was planned, which we acknowledge as a limitation. Future trials should pre-register a sensitivity analysis plan to improve methodological transparency. Future multicenter clinical trials including longer follow-up periods and with different dosages of PENS should be performed.

## 5. Conclusions

In patients with postoperative pain after shoulder arthroscopy, the addition of two sessions of ultrasound-guided PENS targeting the suprascapular and axillary nerves in addition to a manual therapy and exercise program resulted in greater improvements in pain, disability, shoulder-specific function, and short-term local pressure pain sensitivity over the infraspinatus. No additional between-group benefits were observed for widespread pain sensitivity. Limited but statistically significant benefits were observed for some measures of isometric strength and range of motion, although effect sizes were small and clinical relevance should be interpreted with caution. Future confirmatory trials with larger samples, sham controls, and longer follow-up periods are warranted.

## Figures and Tables

**Table 1:** Baseline demographic, clinical, and neurophysiological characteristics of participants by treatment group.

Variables	Manual Therapy/ Exercise	Manual Therapy/ Exercise/Percutaneous Electrical Nerve Stimulation (PENS)
Gender (male/female) *n* (%)	22 (62.9%)/13 (37.1%)	23 (65.7%)/12 (34.3%)
Age (years, SD)	49.0 ± 15.0	51.0 ± 15.5
Weight (kg, SD)	76.0 ± 13.1	79.5 ± 17.5
Height (m, SD)	1.70 ± 0.1	1.75 ± 0.1
BMI (kg/m^2^, SD)	25.6 ± 3.5	26.7 ± 4.7
Type of Surgery *n* (%) Shoulder Instability Rotator Cuff Repair	14 (40.0%) 21 (60.0%)	15 (42.9%) 20 (57.1%)
Affected Side *n* (%) Right side Left side	23 (65.7%) 12 (34.3%)	22 (62.9%) 13 (37.1%)
**Clinical Variables**		
Pain Intensity (NPRS, 0–10 scale, SD)	5.26 ± 2.21	5.58 ± 1.92
Pain-Related Disability (DASH, 0–100 scale, SD)	62.91 ± 25.45	72.78 ± 19.50
Function (SPADI, 0–100, scale SD)	68.85 ± 28.19	79.72 ± 25.81
**Neurophysiological Variables**		
PPT Infraspinatus Muscle (kg/cm^2^, SD)	4.31 ± 1.69	4.67 ± 1.58
PPT Deltoid Muscle (kg/cm^2^, SD)	4.16 ± 1.74	4.28 ± 1.68
PPT Tibialis Anterior (kg/cm^2^, SD)	7.16 ± 2.64	8.35 ± 1.81
**Functional Variables**		
**Isometric Maximum Strength (N)** Shoulder Flexion, (SD) Shoulder Extension, (SD) Shoulder Internal Rotation, (SD) Shoulder External Rotation, (SD) Shoulder Abduction, (SD) Shoulder Adduction, (SD)	34.68 ± 21.67 45.56 ± 24.62 47.65 ± 27.07 36.40 ± 25.18 39.84 ± 29.65 39.23 ± 25.57	24.65 ± 13.27 33.01 ± 16.94 34.42 ± 20.83 26.25 ± 15.15 26.06 ± 15.27 30.58 ± 17.53
**Range of Motion (degrees)** Shoulder Flexion, (SD) Shoulder Extension, (SD) Shoulder Internal Rotation, (SD) Shoulder External Rotation, (SD) Shoulder Abduction, (SD)	121.65 ± 26.34 56.03 ± 14.03 49.00 ± 20.75 34.41 ± 21.06 112.38 ± 36.39	111.94 ± 17.62 63.50 ± 9.54 44.08 ± 22.27 31.67 ± 20.91 116.42 ± 22.89

**Table 2:** Changes in pain intensity, disability, and shoulder-specific function over time by treatment group.

Outcome Group	Pre- Intervention	Post- Intervention	1 Month	3 Months
Pain intensity (Numerical Pain Rating Scale (NPRS), 0–10 scale, 95% CI)				
Manual Therapy/Exercise	5.26 ± 2.21 (4.49, 6.03)	5.21 ± 2.11 (4.47, 5.94)	2.82 ± 1.57 (2.28, 3.37)	1.59 ± 1.31 (1.13, 2.04)
Manual Therapy/ Exercise/PENS	5.58 ± 1.92 (4.93, 6.23)	3.33 ± 1.76 (2.74, 3.93)	1.97 ± 1.30 (1.53, 2.41)	1.11 ± 1.21 (0.70, 1.53)
Pain-Related Disability (Disabilities of the Arm, Shoulder and Hand (DASH), 0–100 scale, 95% CI)				
Manual Therapy/Exercise	62.91 ± 25.45 (54.03, 71.79)			22.53 ± 13.44 (17.84, 27.22)
Manual Therapy/ Exercise/PENS	72.78 ± 19.50 (66.18, 79.37)			20.44 ± 10.80 (16.79, 24.10)
Function (Shoulder Pain and Disability Index (SPADI), 0–100% scale, 95% CI)				
Manual Therapy/Exercise	68.85 ± 28.19 (59.02, 78.69)			19.91 ± 14.21 (14.95, 24.87)
Manual Therapy/ Exercise/PENS	79.72 ± 25.81 (70.99, 88.46)			18.42 ± 12.56 (14.17, 22.67)

**Table 3:** Between-group comparisons of pressure pain thresholds at local and distal sites across follow-up.

Outcome Group	Pre-Intervention	Post-Intervention	1 Month	3 Months
Infraspinatus Muscle (kg/cm^2^, 95% CI)				
Manual Therapy/ Exercise	4.31 ± 1.69 (3.72, 4.90)	4.45 ± 1.75 (3.84, 5.06)	6.03 ± 1.74 (5.43, 6.64)	6.51 ± 1.54 (5.98, 7.05)
Manual Therapy/ Exercise/PENS	4.67 ± 1.58 (4.13, 5.20)	5.46 ± 1.83 (4.84, 6.08)	6.66 ± 1.56 (6.13, 7.19)	6.95 ± 1.81 (6.34, 7.56)
Deltoid Muscle (kg/cm^2^, 95% CI)				
Manual Therapy/ Exercise	4.16 ± 1.74 (3.55, 4.76)	4.27 ± 1.85 (3.62, 4.91)	6.24 ± 1.96 (5.56, 6.93)	6.50 ± 1.76 (5.88, 7.11)
Manual Therapy/ Exercise/PENS	4.28 ± 1.68 (3.71, 4.85)	5.04 ± 2.04 (4.35, 5.73)	6.74 ± 1.97 (6.07, 7.41)	7.14 ± 2.07 (6.44, 7.84)
Tibialis Anterior Muscle (kg/cm^2^, 95% CI)				
Manual Therapy/ Exercise	7.16 ± 2.64 (6.24, 8.08)	7.29 ± 2.64 (6.37, 8.21)	9.53 ± 1.03 (9.17, 9.89)	9.63 ± 0.77 (9.36, 9.90)
Manual Therapy/ Exercise/PENS	8.35 ± 1.81 (7.74, 8.97)	8.51 ± 1.90 (7.86, 9.15)	9.68 ± 0.82 (9.40, 9.96)	9.67 ± 1.11 (9.29, 10.04)

**Table 4:** Between-group comparisons of isometric shoulder strength across follow-up periods.

Outcome Group	Pre- Intervention	Post- Intervention	1 Month	3 Months
Shoulder Flexion (N, 95% CI)				
Manual Therapy/ Exercise	34.68 ± 21.67 (27.1, 42.2)	35.35 ± 22.84 (27.4, 43.3)	46.55 ± 23.44 (38.4, 54.7)	54.07 ± 26.07 (45.0, 63.1)
Manual Therapy/ Exercise/PENS	24.65 ± 13.27 (20.2, 29.1)	28.89 ± 14.93 (23.8, 33.9)	39.80 ± 14.23 (35.0, 44.6)	46.94 ± 18.09 (40.8, 53.1)
Shoulder Extension (N, 95% CI)				
Manual Therapy/ Exercise	45.56 ± 24.62 (37.0, 54.1)	47.18 ± 24.56 (38.6, 55.7)	51.62 ± 22.18 (43.9, 59.4)	56.15 ± 24.06 (47.8, 64.5)
Manual Therapy/ Exercise/PENS	33.01 ± 16.94 (27.3, 38.8)	36.66 ± 18.02 (30.6, 42.8)	48.87 ± 19.83 (42.2, 55.6)	53.92 ± 22.77 (46.2, 61.6)
Shoulder Internal Rotation (N, 95% CI)				
Manual Therapy/ Exercise	47.65 ± 27.07 (38.2, 57.1)	50.81 ± 30.09 (40.3, 61.3)	62.06 ± 30.14 (51.6, 72.6)	67.86 ± 30.68 (57.2, 78.6)
Manual Therapy/ Exercise/PENS	34.42 ± 20.83 (27.4, 41.5)	40.47 ± 21.94 (33.0, 47.9)	56.90 ± 24.16 (48.7, 65.1)	63.92 ± 28.30 (54.4, 73.5)
Shoulder External Rotation (N, 95% CI)				
Manual Therapy/ Exercise	36.40 ± 25.18 (27.6, 45.2)	37.25 ± 24.82 (28.4, 46.1)	48.41 ± 21.84 (40.8, 56.0)	55.21 ± 25.19 (46.4, 64.0)
Manual Therapy/ Exercise/PENS	26.25 ± 15.15 (21.1, 31.4)	28.42 ± 15.30 (23.2, 33.6)	41.67 ± 15.53 (36.4, 46.9)	47.16 ± 19.24 (40.7, 53.7)
Shoulder Abduction (N, 95% CI)				
Manual Therapy/ Exercise	39.84 ± 29.65 (29.5, 50.2)	39.83 ± 27.68 (30.2, 49.5)	54.40 ± 31.77 (43.3, 65.5)	60.03 ± 29.11 (49.9, 70.2)
Manual Therapy/ Exercise/PENS	26.06 ± 15.27 (20.9, 31.2)	30.73 ± 16.50 (25.1, 36.3)	45.34 ± 20.37 (38.5, 52.2)	53.48 ± 24.79 (45.1, 61.9)
Shoulder Adduction (N, 95% CI)				
Manual Therapy/ Exercise	39.23 ± 25.57 (30.3, 48.2)	39.99 ± 22.93 (32.0, 48.0)	50.54 ± 23.11 (42.5, 58.6)	56.94 ± 25.41 (48.1, 65.8)
Manual Therapy/ Exercise/PENS	30.58 ± 17.53 (24.7, 36.5)	34.86 ± 18.21 (28.7, 41.0)	52.61 ± 23.20 (44.8, 60.5)	58.22 ± 27.69 (48.9, 67.6)

**Table 5:** Changes in shoulder range of motion over time by treatment group.

Outcome Group	Pre- Intervention	Post- Intervention	1 Month	3 Months
Shoulder Flexion (degrees, 95% CI)				
Manual Therapy/ Exercise	121.65 ± 26.34 (112.5, 130.8)	127.71 ± 27.13 (118.2, 137.2)	145.74 ± 21.65 (138.2, 153.3)	166.38 ± 15.50 (161.0, 171.8)
Manual Therapy/ Exercise/PENS	111.94 ± 17.62 (106.0, 117.9)	123.97 ± 17.54 (118.0, 129.9)	149.11 ± 20.74 (142.1, 156.1)	166.56 ± 17.39 (160.7, 172.4)
Shoulder Extension (degrees, 95% CI)				
Manual Therapy/ Exercise	56.03 ± 14.03 (51.1, 60.9)	57.82 ± 13.38 (53.2, 62.5)	67.91 ± 6.08 (65.8, 70.0)	70.24 ± 2.74 (69.3, 71.2)
Manual Therapy/ Exercise/PENS	63.50 ± 9.54 (60.3, 66.7)	66.03 ± 6.81 (63.7, 68.3)	70.00 ± 0.00 (70.0, 70.0)	70.00 ± 0.00 (70.0, 70.0)
Shoulder Internal Rotation (degrees, 95% CI)				
Manual Therapy/ Exercise	49.00 ± 20.75 (41.8, 56.2)	52.85 ± 21.16 (45.5, 60.2)	67.91 ± 6.08 (65.8, 70.0)	70.24 ± 2.74 (69.3, 71.2)
Manual Therapy/ Exercise/PENS	44.08 ± 22.27 (36.6, 51.6)	50.03 ± 22.41 (42.5, 57.6)	68.75 ± 14.66 (63.8, 73.7)	70.00 ± 0.00 (70.0, 70.0)
Shoulder External Rotation (degrees, 95% CI)				
Manual Therapy/ Exercise	34.41 ± 21.06 (27.1, 41.8)	39.15 ± 22.40 (31.3, 47.0)	63.24 ± 19.19 (56.5, 69.9)	72.74 ± 16.70 (66.9, 78.6)
Manual Therapy/ Exercise/PENS	31.67 ± 20.91 (24.6, 38.7)	37.11 ± 21.64 (29.8, 44.4)	63.44 ± 19.72 (56.8, 70.1)	78.64 ± 16.85 (73.0, 84.3)
Shoulder Abduction (degrees, 95% CI)				
Manual Therapy/ Exercise	112.38 ± 36.39 (99.7, 125.1)	118.06 ± 36.59 (105.3, 130.8)	146.38 ± 22.97 (138.4, 154.4)	164.91 ± 20.33 (157.8, 172.0)
Manual Therapy/Exercise/PENS	116.42 ± 22.89 (108.7, 124.2)	126.28 ± 21.60 (119.0, 133.6)	155.97 ± 19.10 (149.5, 162.4)	170.83 ± 15.55 (165.6, 176.1)

## Data Availability

All data derived from this study are presented in the text. Data are available from the corresponding author on proper request.

## Supplementary Materials

The following supporting information can be downloaded at: https://www.mdpi.com/article/10.3390/healthcare14111471/s1, Table S1: Multimodal evidence-based manual therapy program including joint mobilizations, soft-tissue techniques, NMES, strengthening, endurance, and proprioceptive exercises; Figure S1: G*Power 3.1 output for the a priori sample size calculation based on a repeated-measures ANOVA (within–between interaction) with α = 0.05, power = 0.95, effect size f = 0.25, 2 groups, and 6 measurement points, yielding a minimum required total sample size of 28 participants (actual power = 0.954).
