# Supplementary material for: Ultrasound-Guided Percutaneous Electrical Nerve Stimulation (PENS) as an Adjunct to a Multimodal Physical Therapy Program for Postoperative Shoulder Pain: A Randomized Clinical Trial
Source: Healthcare (Basel). 2026 May 26;14(11):1471. doi: 10.3390/healthcare14111471 (PMC13257376; doi:10.3390/healthcare14111471)

Ultrasound-Guided Percutaneous Electrical Nerve Stimulation (PENS) as an Adjunct to a Multimodal Physical Therapy Program for Postoperative Shoulder Pain: A Randomized Clinical Trial Supplementary Materials

Supplementary Table S1: Multimodal evidence-based manual therapy program including joint mobilizations, soft-tissue techniques, NMES, strengthening, endurance, and proprioceptive exercises.

**Phase 1** (weeks 1–4)
Passive mobilization of the shoulder (pain-free).
Active-assisted and active range-of-motion therapy within pain-free range.
Soft-tissue mobilization techniques for the shoulder musculature.
Isometric contractions of the shoulder musculature.
Neuromuscular electrical stimulation (NMES).
Cryotherapy.
* Sling use was maintained between sessions according to medical prescription.
**Phase 2** (weeks 6–7)
Progressive active range-of-motion therapy within pain-free range.
Soft-tissue mobilization techniques for the shoulder musculature.
Pain-free isotonic strengthening exercises of shoulder muscles/scapular stabilizers.
NMES targeting deltoid and infraspinatus activation.
**Phase 3** (weeks 8–10)
Active mobility within pain-free range of motion.
Soft-tissue mobilization techniques for the shoulder musculature.
Endurance exercises for shoulder muscles and scapular stabilizers.
Proprioceptive and scapulohumeral stability training.
**Phase 4** (weeks 8–12)
Increasing-load strengthening and endurance exercises for shoulder muscles and scapular stabilizers.
Neuromuscular control and proprioceptive training.

Supplementary Figure S1: G*Power 3.1 output for the a priori sample size calculation based on a repeated-measures ANOVA (within–between interaction) with $\alpha = 0.05$, power = 0.95, effect size f = 0.25, 2 groups, and 6 measurement points, yielding a minimum required total sample size of 28 participants (actual power = 0.954)

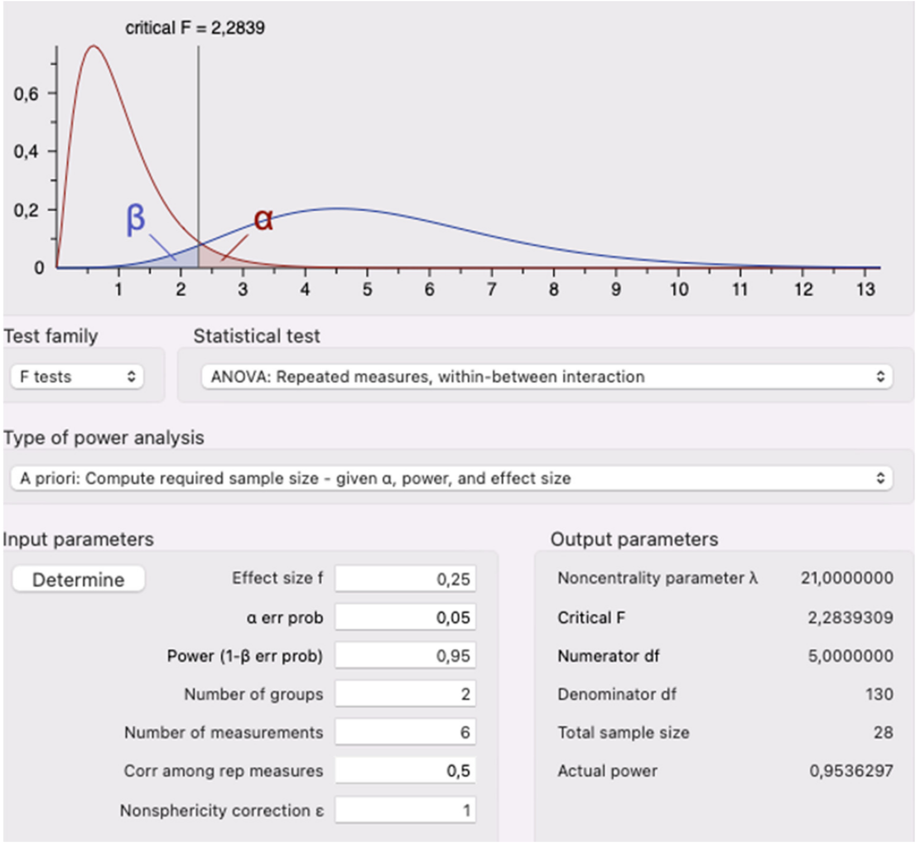

Supplement: Supplementary file 1 [file healthcare-14-01471-s001.zip › healthcare-4215675-supplementary.pdf]
